# Negotiating boundaries of care: an interpretative phenomenological analysis of the relational conflicts surrounding home mechanical ventilation following traumatic spinal cord injury

**DOI:** 10.1080/21642850.2018.1462708

**Published:** 2018-04-25

**Authors:** A. Dickson, T. Karatzias, A. Gullone, G. Grandison, D. Allan, J. Park, P. Flowers

**Affiliations:** a School of Health & Life Sciences, Glasgow Caledonian University, Glasgow, UK; b School of Health and Social Care, Edinburgh Napier University, Edinburgh, UK; c School of Health and Social Sciences, University of Edinburgh, Edinburgh, UK; d Queen Elizabeth National Spinal Injuries for Scotland, Southern General Hospital, Glasgow, UK

**Keywords:** Mechanical ventilation, spinal cord injury, qualitative, IPA, carer, negotiating boundaries

## Abstract

**Objectives:** The aim of this study is to explore the phenomena of mechanical ventilation following traumatic spinal cord injury from three simultaneous perspectives; patients who require full-time mechanical ventilation (*n* = 8), their informal family carers (*n* = 8) and their formal carers (*n* = 11). We focus upon the intra and inter- personal challenges of establishing boundaries within the triad.

**Design:** Qualitative study.

**Methods:** Semi-structured interviews were transcribed verbatim and analysed using interpretative phenomenological analysis (IPA). In order to encapsulate the inter-subjective, multi-dimensional and relational aspects of the experience, we focussed on recurrent themes which were independently reported across all three participant groups.

**Results:** One major inter-connected recurrent theme was identified: 1) ‘Negotiating boundaries of care and finding a “fit”’. It centres around establishing a ‘line’, or a boundary, which was imperative for retaining a sense of independence (for patients), a sense of home and privacy (for informal carers) and difficulties balancing complex care provision with the needs of family members so as not to cross that ‘line’ (for formal carers).

**Conclusions:** The findings highlight the need for focussing on a ‘fit’ within the triad, balancing boundaries of care in order to establish a productive, satisfactory psycho-social environment for all three participant groups to live and/or work within. Recommendations for both future care provision and future research are suggested.

## Introduction

Home Mechanical Ventilation (HMV) has been described as one of the most revolutionary and intricate forms of medical treatment provision beyond the hospital environment (Glass, Grap, & Battle, 1999; Lewarski & Gay, 2007). Patients typically require highly specialised care which comprises 24 h monitoring and support. The number of individuals requiring HMV following traumatic injury (e.g. spinal cord injury), is increasing. This is, in part, due to improvements in the development of mechanical ventilators themselves but also, in part, due to advances in treatment and medical practice. The use of HMV is only expected to increase due to the need for cost-effective care and the documented benefits for patients. For example, HMV has been reported to improve patient quality of life and prolong life (Lloyd-Owen et al., 2005).

HMV brings about physical, psychological and social change (Lewarski & Gay, 2007) and patients often experience loneliness, sadness, hopelessness, anergia, anxiety, insomnia and fear (Gleckman, Phillippi, & Rohr, 1996) in addition to communication difficulties. This can result in patients feeling ‘trapped in a silent world’ (Carroll, 2007). Due to the significant impairment associated with spinal cord injury (SCI), the patient becomes wholly dependent on others to meet practically all of their basic needs. This often results in diminished quality of life but this can be minimised by good quality relationships with others (Charlifue et al., 2011).

Family members often assume an informal carer role and become actively involved in both personal and technical care provision (Kirk & Glendinning, 2002). Emotional and social burden, stress, anxiety, depression and an overwhelming sense of responsibility are well documented in the caregiver literature (Choi, Donahoe, Zullo, & Hoffman, 2011; Van Pelt et al., 2007). Family carers also experience a decline in their own health over time (Weitzenkamp, Gerhart, Charlifue, Whiteneck, & Savic, 1997) and report difficulties in dealing with formal carers, insurance coverage, equipment suppliers and a lack of preparation for the carer role (Findeis, Larson, Gallo, & Shekleton, 1994).

For family members who have a child requiring HMV, intimidation and vulnerability as a parent are common and feelings of intrusiveness within the former private, home environment are reported as they adapt to their medicalised surroundings (Wang & Barnard, 2008). Distress and frustration are commonplace as choice, freedom and privacy diminish as the formal care team enter the home (Coffman, 1995; Wang & Barnard, 2008). Role ambiguity (Morse, Wilson, & Penrod, 2000), social isolation and inadequate support are also noted (Beale, 2002). A lack of understanding of what care provision entails within the home environment can result in fragmented collaborations between the formal care team and informal, family carers (Wang & Barnard, 2008). This has potential to negatively impact psycho-socially on the patient, family caregiver and formal care team.

From the formal carer’s perspective, they ultimately become a ‘guest’ or a ‘professional’ in the patient’s home but they report that it is unattainable to be both at the same time (Oresland, Maatta, Norberg, Jorgensen, & Lutzen, 2008). Attempting to be ‘guest’ can create obstacles to clear communication between the formal carer and the patient and/or their family member (Dybwik, Nielsen, & Brinchmann, 2011). Nurses, for example, often feel their sense of authority is challenged when family members also provide specialist care (Kirk & Glendinning, 2002) and report strategies such as keeping a low profile, maintaining a neutral demeanour, and ‘holding their tongues’ in order to avoid inter-personal conflicts. Indeed, many health care providers report that dealing with the patient’s family, is the most challenging aspect of HMV care provision (Dybwik et al., 2011). High turnover rates in formal carers are common and only add further to dysfunctional relationships between family and formal carers (Patterson, Jernell, Leonard, & Titus, 1994). Consistency of care, therefore, is essential to optimise the quality of the care environment (Ballangrud, Bogsti, & Johansson, 2009).

It would appear, therefore, that successful adaptation and positive psycho-social outcomes in HMV, depend partly on the interpersonal relationships and communication between the patient, family carer and formal care team. There remains a paucity of research exploring the relational aspects of HMV and the intra- and inter-personal challenges that exist within the care triad- that is, the patient, the informal carer and the formal care team. The majority of work in this area has focussed on: 1) the patient’s perspective of becoming dependent on a ventilator (Dreyer, Steffensen, & Pedersen, 2010; Lindahl, Sandman, & Rasmussen, 2005), meaning of life (Lindahl, Sandman, & Rasmussen, 2003), quality of life (Charlifue et al., 2011; Noyes, 2006) and 2) parental carers (Dybwik et al., 2011; Lindahl & Lindblad, 2011). The current study is the first to employ a multi-perspective approach to the relational aspects of HMV. We do so, within the spinal cord injury (SCI) population. Given the originality of this study, we employed Interpretative Phenomenological Analysis- an inductive, idiographic method of inquiry which seeks to explore the lived experience of the phenomena under investigation. The aim was to capture the meaning of HMV, from the perspective of the research participants themselves. This is essential if we are to develop effective interventions which will improve the psycho-social environment of those living and working with HMV in the future.

## Methods

### Participants

Participants (*n* = 27) included a) patients requiring full-time mechanical ventilation following traumatic spinal cord injury (*n* = 8); b) informal (family/spousal) carers (*n* = 8); and c) formal (paid) carers (*n* = 11). Patients were seven males and one female, aged 18–71 years (mean 44.3 years). The duration of time dependent on mechanical ventilation ranged from 4–20 years, with seven patients living within their home and one in a residential care home. Informal carers were seven females and one male, aged 39–70 years (mean 51.1years). Three were siblings of the patient, three were spouses and two were mothers. Formal carers were ten females and one male, with the number of years caring for patients requiring mechanical ventilation ranging from 1–21 years. Further details of participants are shown in Table 1:

**Table 1.** Participant information.

Patient pseudonym	M/F	Age (yrs)	Time dependent on MV (yrs)	Time since injury (yrs)	Place of residence	Informal carer pseudonym	M/F	Age (yrs)	Relationship to patient	Formal Carer pseudonym(s)	Experience of MV carer role (yrs)
Colin	M	51	5–6	27	Family home	Linda	F	45	Sister	Lorna	1–2
Jane	F	71	4	3–4	Care home	Steve	M	71	Husband	Cathie & Sylvia	3, 2
Richard	M	35	20	20–21	Home, living independently	Aileen	F	39	Sister	Karen & Gillian	20, 21
William	M	31	5–6	7	Family home	Sharon	F	55	Mother	Morag	5
Craig	M	60	6–7	34	Home, living independently	Kaye	F	49	Sister	David	2
Robert	M	18	Phrenic Nerve Pacing	15.5	Family home, University (on campus residence)	Lynn	F	50	Mother	Lisa	10
Roy	M	50	8	7	Family home	Rachel	F	50	Wife	Ruth	8
John	M	38	10	10	Family home	Susan	F	50	Wife	Irene & Lorna	10, 2

Patient inclusion criteria included: a) adults over the age of 18 years; b) entirely dependent on the mechanical ventilator (requiring a minimum of 12 h ventilation per day); c) fluent in English (for the purpose of the interview); d) sustained a traumatic spinal cord injury; e) at least 12 months post spinal cord injury; and f) able to give verbal and/or written consent. Carer inclusion criteria included: a) adults over the age of 18 years; b) fluent in English; c) able to provide written consent; d) known the patient for at least 6 months prior to the spinal cord injury (informal carers); e) provide regular care to the patient (informal and formal carers).

### Procedure and interview

Recruitment occurred via the Clinical Director at the Queen Elizabeth National Spinal Injuries Unit for Scotland (QENSIUS), Southern General Hospital (Glasgow). The Clinical Director identified potential participants who met the inclusion criteria via the hospital database to generate a list of all full-time mechanically ventilated patients. The Respiratory Nurse (providing routine care to all patients on the list) then approached potential participants to provide an overview of the research project, including its aims and what would be required in partaking. Those participants who were willing to take part provided verbal consent for their contact details to be passed on to the Research Assistant. The Research Assistant then contacted the potential participants to provide further information, answer any questions they may have had and arrange a date and time for the interviews to commence. Interviews were conducted during a routine care visit with the Respiratory Nurse. This was an ethical requirement of the QENSIUS and ensured the safety of the researcher. Interviews were conducted by the third author, a highly experienced qualitative researcher with extensive interview experience. Interviews with each member of the triad (e.g. the patient, the informal carer and the formal carer) were conducted on the same day (for geographical reasons) but all participants were interviewed separately and in private. An information leaflet was read to all participants prior to consent being taken and another opportunity for questions was provided at this time. All participants were assured that the data would remain anonymous (pseudonyms would be given to all participants) and confidential, that they could withdraw from the study at any time and that should they decide not to partake, it would not affect their care in any way.

NHS ethical approval and University ethical approval was sought prior to the commencement of the study. Interview schedules were designed prior to the interviews which comprised open-ended, non-directive questions such as ‘Tell me about your experience of mechanical ventilation’, ‘What is a typical day for you?’ and ‘What impact has mechanical ventilation had on your life?’ However, the schedules were not followed in a strict way; instead, a process of reflecting (‘You said there that … ’) and probing (‘what did you mean by that?’) was adopted. This ensured that the content of the interview was directed by the participant. Clarification was sought throughout by the Research Assistant to ensure that they were interpreting the participant’s account appropriately. The interviews lasted for approximately 1 h. Pseudonyms were given to all participants and the interviews were transcribed verbatim and subjected to Interpretative Phenomenological Analysis (IPA).

### Analysis

The first and last authors took the lead on analysis, manually analysing all of the transcripts independently but the fourth author also provided credibility checks (e.g. ensuring coding was appropriate). The process of analysis involved several key stages, as suggested by Smith, Flowers, and Larkin (2009). We began by analysing each of the three groups of participants separately. For the first group (patients), each interview was read several times to increase familiarity with the participant’s narrative. The ﬁrst phase of the analysis highlighted key words, phrases and idiosyncratic ﬁgures of speech. The transcript was then interrogated further by making comments and suggestions or asking questions in an attempt to generate ‘meaning’ grounded in the participant’s own words. From here, initial themes were identiﬁed. Relationships and tensions between codes were sought out and those which appeared to refer to similar issues were grouped together as themes. From this analysis, recurrent themes emerged which were those evident in more than half of the accounts. The coding that refers to these themes was then reviewed to ensure the themes remained relevant at the broader level, without compromising the meaning or signiﬁcance of the participants’ comments. Any individually coded items not relevant were removed from the broader themes. These broad themes themselves were then revised further. Quotations from participants were reviewed with reference to the themes, to ensure that the themes reﬂected what the participants were saying before all of the transcripts were re-read to ensure that the themes were accurate in relation to the global experiences of the participants. If it was felt that a mismatch was evident, the terminology, speciﬁcity and focus of the themes were revisited until the descriptive titles and the overall testimonies were congruent. This cyclical process was continued until no further clariﬁcation or reﬁnement was found. The procedure was then replicated for both of the carer groups in turn until a final list of recurrent themes had been generated for each group. To complete the analytic process, we then created a table of Master themes across all three groups (see Table 2). The aim was to explore the commonalities of issues across the three groups in order to capture the intra-subjective perspectives of the intersubjective, multi-dimensional and relational aspects of living and working with mechanical ventilation following a spinal cord injury.

**Table 2.** Master themes across all three participant groups.

Master Theme	Patient	Informal (family) caregiver	Formal (paid) caregiver
Acceptance and adjustment	yes	yes	no
Care regime	no	yes	yes
Concerns for children	no	yes	no
Coping	yes	yes	no
Dehumanisation	yes	no	no
Family home vs residential care	no	yes	no
Fears for the future	no	yes	no
Inconsistent training	no	yes	yes
Learning on the job	no	no	yes
Loss of control	yes	yes	no
Loss of freedom	yes	yes	no
Loss of independence	yes	yes	no
Machinery- unpredictability	no	yes	yes
Negotiating boundaries of care and finding a ‘fit’	yes	yes	yes
Onset of injury/realisation of severity of injury	yes	yes	no
Poor medical care/lack of training	no	yes	yes
Rewarding nature of caregiving	no	no	yes
Stigma	yes	no	no
Suicidality	yes	no	no
Ups and downs	yes	yes	no

**Figure 1.**
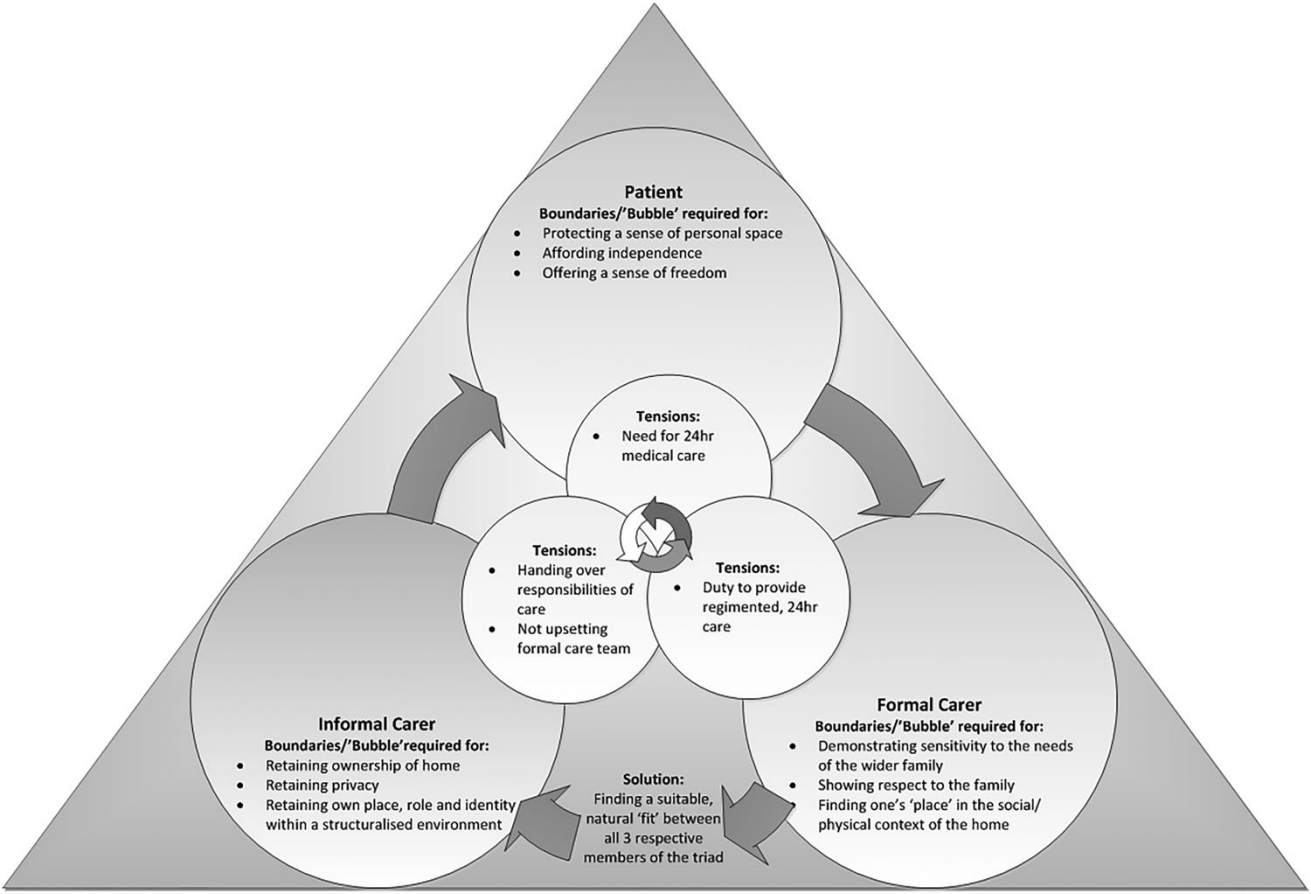
Negotiating boundaries of care and ‘finding a fit’.

Therefore, this article centres on a single Master theme, which was independently identified as recurrent themes across all three participant groups: ‘Negotiating boundaries of care and finding a “fit”’. Figure 1 provides a visual overview of the dynamics between the three participant groups, in relation to this particular Master theme:

## Ethics Statement

Ethical approval was confirmed from both Edinburgh Napier University and the appropriate NHS ethics committees prior to the study commencing. All participants provided written consent for their participation in the study and have been anonymised using pseudonyms throughout.

## Results

### Negotiating boundaries of care and finding a ‘fit’

All three participant groups highlighted complexities in negotiating boundaries of care within the home environment. Each group highlighted the need for boundaries, or lines to be drawn, which preserved cherished meanings and roles within a complex emotional, relational and technological setting. For the patients, these boundaries centred upon the tension between their acknowledged need for constant and ongoing practical and physical aspects of care but their equal need for personal space, and a sense of independence and freedom. For the informal carers, boundary work focussed upon the challenge to retain a sense of ‘home’ in what had become a regimented medicalised environment. Informal carers also wanted to retain some sense of privacy in an environment requiring the presence of a 24-hour care team and an acknowledgement of their own place as an informal carer within that structured, professional care environment. The formal carers also acknowledged the need to navigate the balancing of complex, regimented provision of care to the patient with sensitivity to the needs of their family. Ultimately, what emerged from the analysis of all three participant groups was the importance of finding a suitable ‘fit’ between the patient, the family and the formal care team in relation to how the set their respective boundaries. We will now discuss the intricate intra and inter-personal needs of each participant group in turn.

#### ‘There is a line’: the patients’ perspective

The patients were entirely dependent on their care team, who were required to be present 24 h a day to manage the complex and unpredictable nature of their condition and the ventilator. For the patients, the notion of identifying a boundary between them and the care team was essential for the preservation of some sense of independence. Many of the participants reported that such constant care could be ‘stifling’ and there was a need for opportunities where they could feel that the ‘shackles were off’. This concept of independence was particularly difficult to define and achieve:

I’m dependent on them [carers] to give me my independence. But to have my independence, I have to maintain independence from that [ventilator] (John)

The patients suggested that while the care team were required to be physically present at all times, their proximity to the patient and the level of engagement with them could ideally be controlled according to their own needs. Many participants highlighted a need to remain independent of the ‘influences of the many coming and going personalities of the care team’ and suggested that a ‘line’ of care should not be crossed in order for them to maintain some sense of independence:

There is a line, isn’t there, where you cross where it’s business and where it’s personal, that was a big problem in the early days (John)

Like John, many of the participants held expectations of clearly defined boundaries of their care. Here, it is as though John seeks affirmation that carers crossing the ‘line’ was inappropriate, unacceptable and that he was not unrealistic to expect them to respect this line. Although many of the participants suggested that for the care team, establishing and adhering to this ‘line’ was difficult, some of the participants spoke of introducing the concept of a ‘care bubble’ with its associations of transparency and mobility balanced with a robust boundary and demarcation. Here, there is an analogy where the care team can step into the ‘care bubble’ to deliver care (business) and then step out again when required (to safeguard personal space). This need for the care team to ‘retreat into the background’, was considered important for patients in terms of the preservation of their independence:

You have the separate realms of the client and obviously, the disabled person but you know, the client leading his or her life separately. Well, I say separately, but you know, as themselves and the carers popping in and out where that’s necessary (Robert)

Robert’s extract above again highlights a need to define clear boundaries between the patient and the care team- a need for the patient to be distinctly unconnected to the carers- to be independent, separate and to be ‘them self’. William also highlights how this can be achieved by using the example of the care team (as waiters) in a restaurant (resembling the care home):

[waiter/carer] Comes in when needed, serves the food and then disappears, because they aren’t, so to speak, directly part of the family engagement with the meal, but are necessity to it happening, if you like (William).

#### ‘My home is not my home anymore’: the informal carers’ perspective

There were a number of difficulties facing the informal carers when the formal care team arrived. In the context of a small group of people sharing the same domestic space, family structures, roles and concomitant boundaries were all challenged. For example, the arrival of the care team posed: 1) an expectation of immediately ‘handing over’ all care to the care team; 2) a threat to their sense of ownership of their home; and 3) a loss of privacy. Underpinning all of the personal and relational challenges presented above, was the apparent brash and presumptuous entry of the formal care team to the family home. Many of the participants were, themselves, the primary carer for the patient prior to the appointment of the formal care team. The latters’ arrival sparked a sudden realisation of the transition in informal carers’ former identities and carer roles. Consequently, many of the informal carers struggled to simply ‘hand over’ the care of their loved-one automatically to a group of complete strangers:

Suddenly you were expected to draw back which was difficult but added to that was the fact that I was having to try and draw back from caring, the personal care as well, you know, watching people doing things where you want to go and do it yourself. So it was a really, really hard thing to learn and I had a lot of emotion, really struggled a lot, you know, time has taught me to trust the team (Sharon).

The entry of the formal care team to the family home appeared to be threatening and invasive for the informal carer and it occurred at a time when they were particularly vulnerable emotionally- the cherished roles and relationships that they previously shared with patients were now changed forever. At this pivotal point of relationship change, the arrival of the formal care team symbolised a further proliferation of threat in terms of the informal carer’s role and identity more broadly. All but one of the informal carers spoke of the sudden and drastic change in the home environment upon the arrival of the care team:

They [formal carers] were so invasive when they first started. They didn’t see it as a home. They saw it as a kind of NHS [National Health Service] type building. So, the whole house was like … . And they were … and it was their domain, their workplace. Rather than it being … it was their workplace first and our home second (Susan)

The above extract highlights a perceived attack on Susan’s personal space and the depersonalisation of the home environment. There is a feeling here of the home as being carer ‘territory’, a medicalised environment now as opposed to the safe haven of her former home. Many of the informal carer participants reported a similar loss of control over their own personal space, with some reporting that they now ‘never had a bit of peace’. Participants also reported formal carers overstepping the ‘line’ on a more personal, private level:

They thought they were part of the family so there was no … they wanted to be best buddies. Some of them were buying things for the kids, presents, on a regular basis, or trying to get them to go next door and do crafts with them and that’s my job (Susan)

Within the context of a threatened home environment, Susan introduces a parallel threat to her perceived role and status within the family home. Similarly Lynn describes feeling usurped:

There was one care member in particular who I felt was trying to take my place (Lynn)

In response, many of the informal carers reported setting clear boundaries with the formal carers, in a bid to regain control of their homes and prevent them from overstepping the ‘line’ in future. The desire to preserve and define the family through boundaries (both in a physical and relational sense) is illustrated with the clear demarcation of pronouns (‘us’ vs ‘them’) in the extract below. This extract shows, for Rachel, the primacy of maintaining the family’s integrity:

Please don’t walk through without letting us know you’re coming, they would get offended. It’s like ‘how dare you tell me’ whereas I felt that this is my home, this is my house, that kind of thing. And that was again; I could see it from both sides but it was tearing the family apart (Rachel)

Many of the participants reported that the formal care team took the setting of such boundaries ‘personally’. Small disagreements then caused tension- small requests ‘grew arms and legs’. While the setting of boundaries afforded the informal carers an opportunity to regain some control and privacy in their own homes, they also came with concerns: upsetting the care team could compromise the care provision for the patient:

If you started to draw clear boundaries and started to upset the team that were caring for him [patient], there was a risk that they would withdraw the quality of the care that they were offering him (Susan)

There appears to be recognition here of the need to negotiate acceptable, clear boundaries in a sensitive and tactful manner. Interestingly, there is an essence of informal carers being fearful of breaching formal carer boundaries when attempting to set their own limits. This appears to be a very fragile process and concerns of whether these two sets of boundaries may be compatible peppers much of the informal carer accounts. It is also interesting that the informal carers ‘walking on egg shells’ accounts (so as not to upset or offend the care team) juxtapose with the brash manner in which the formal care team were reported to behave within the family home.

#### ‘This is my house and you can’t come past the door’: the formal carers’ perspective

Many of the formal carers were aware of the ‘line’ that had been set by both the patient (in terms of independence) and the informal carers (in terms of privacy and ownership of the home). They highlighted a struggle to ‘find their place’ within the social and physical context of the family house:

The difficulties were, I suppose in a way, trying to find your role, to try and find a neutral place within the family setting but still deliver the standard of care without … do you know, it’s basically just knowing your place. That’s exactly what it is. I’m not going to posh it up anymore. I’m just going to say it how it is. It’s just knowing your place and it’s just really important (Lisa)

Lisa’s frank and pithy account brings to light the challenge of accepting her position purely as a carer within the family home and remaining impartial and unbiased at all times. There is also an acknowledgement that the informal carer has authority here, despite the formal carers having charge over patient care. There is also a realisation that formal carers have to be respectful and mindful of that power relation at all times.

Negotiations over boundaries with the informal carer were particularly challenging for formal carers. Participants reported that the informal carers ‘guarded their privacy’ and many formal carers were faced with resentment and resistance in taking up their role:

This is the family home. They didn’t want us here … It was made perfectly clear to everybody that started at the beginning that … I’d like to say it as seen and not heard. You’re there to do a job, they don’t want to be your best friends. They didn’t hire you to be best friends (Lisa)

The above extract is peppered with a sense of rejection and exclusion. The quote is tinged with a sense of being reprimanded for stepping over the ‘line’. There is also an element of down-ranking -‘seen and not heard’ evoking the role of the servant and perhaps suggests that they had been admonished in the past. The awareness of set boundaries for privacy was also described in relation to other family members living within the home. Below, Lorna and Morag discuss the same dynamic in different homes. While Lorna highlights the irregular and problematic nature of these boundaries, Morag brings to light the impact of them on the care team :

We were not to be involved with the children in any way, shape or form and that’s sometimes very difficult … was difficult because if we were asked to go through the house we weren’t even allowed to speak to them, to say hello or anything and was very unnatural (Lorna)

We couldn’t say or do anything, you know, we’d … on a nightshift you were practically tiptoeing about. It was uncomfortable at first. At least half of the team suffered for a couple of years and left (Morag).

## Discussion

In this article, we have presented a multi-perspective account of the intra- and inter-personal tensions within the care triads. Problems within the triad can have a negative psycho-social impact on all three participant groups. This research adds to the growing body of literature on HMV in a number of ways: 1) it is novel in its multi-perspective highlighting relational aspects within the care triad; 2) it is unique in its focus on a specific patient group (SCI); 3) it is original in including the perspectives of a range of informal and family carers; and 4) it is the first study of its kind to do so using IPA in a traditional way to firstly explore experiences from the participants individual perspectives and then subsequently through an overall analytical perspective addressing the ways in which key themes within the diverse participant groups coalesce.

Previous research on mechanically ventilated patients has reported loneliness, sadness, hopelessness, anergia, anxiety, insomnia and fear (Gleckman et al., 1996) in addition to communication difficulties (Carroll, 2007; Khalaila et al., 2011). While our study also explored communication difficulties, the participants focussed on inter-personal conflict with the carers. For patients, tension centred on a dependency on carers to afford them the luxury of a sense of independence. This was especially difficult to achieve given the often overwhelming extent of the care provision. Patients identified a ‘line’ where business-related caregiving activities existed on one side, and more personal, private activities existed on the other. Participants accepted the presence of formal carers to provide necessary business-related caregiving duties. However, they desired carers’ presence to remain in this domain as far as possible- with as little existence on the other side of the line, as possible. Some participants introduced the concept of the ‘care bubble’, where the formal care team could ‘step in’ to the bubble to provide necessary care but should ‘step out’ of the bubble when the patient required privacy and independence. This was proposed by the patients, to allow them to alter the level of dependency as far as possible and was described by Robert as an ‘extreme concept to function fully’. However, this would only be effective if clear boundaries were identified, agreed upon and adhered to. The bubble metaphor is, in itself, worth noting- perhaps highlighting the transparency of the patient’s life (and physical care needs) and yet at the same time, the invisibility of their psychosocial needs. The care bubble may emphasise the fragility of the patient and perhaps the care boundary itself. The metaphor emphasises that a ‘line’ must be drawn and adhered to on all parts in order to prevent the bubble from bursting.

Informal carers reported the intra- and inter-personal challenges associated with the transition from a personal, private home to a medicalised environment. Informal carers experienced a sense of loss, vulnerability and intimidation within the context of their new medicalised home. This was particularly characterised by feelings of diminishing privacy- they didn’t get ‘a minute’s peace’. There was a feeling that the formal care team had invaded their home and were, at times, disrespectful, rude, and usurping cherished roles. To return to the ‘bubble’ metaphor, it is possible that informal carers felt that their ‘bubble’ had been burst- their previously private, former intimate relationships and roles had been fractured or disintegrated by the introduction of the formal care team. We also consider whether the creation of the patient-formal carer ‘bubble’ left informal carers feeling excluded or rejected in some ways- it is as though they are now on the outside of the bubble- looking in. Alternatively, perhaps they no longer have a ‘bubble’ to protect their private, intimate relationships. This often led to fragmented collaborations and tension within the home/care environment. While these findings have been reported elsewhere in parental carers in HMV (Wang & Barnard, 2008) our results indicate these feelings are evident in the wider, informal carer population (as previously reported by Morse et al., 2000). While we acknowledge that moderate conflict may be natural, and that such conflict is unlikely to be entirely resolved (due to changes to previous roles), our study identifies the role that ambiguity plays in creating tension in the care environment. Informal carers tended to ‘drip feed’ their feelings to formal carers, often in moments of heated frustration. While this was important for the informal carers to regain some control, a few were then concerned that it would affect the quality of the care provision for the patient (e.g. Susan). Such fragmented communication and lack of clear boundaries within the care environment had a negative psychosocial impact on all three members of the triad and only contributed further to role ambiguity in both informal and formal carers.

The informal carers in the current study also highlighted difficulties in ‘handing over’ care responsibility to the formal care team. Many of the informal carers were trained in HMV care prior to the patients discharge and they were a central and necessary part of the care team for a time until a more formal, permanent care team could be put in place. This meant that the arrival of the formal care team was especially challenging- in addition to the feelings outlined above, informal carers were all of a sudden no longer required. They reported an expectation that they would automatically withdraw care but experienced anxieties in a group of strangers then taking charge of their loved ones. Issues of trust were highlighted and it was only over time (and through transparent, open communication) that trust was eventually established and anxieties gradually subsided. Developing reciprocal trust has been reported as a basic and central process facilitating the patient-carer relationship (Goldstein et al., 1998) but it also appears central at the triadic level, as opposed to simply a dyadic one.

Formal carers also discussed inter-personal tensions within the care environment. Challenges for formal carers included balancing the complex, intricate care needs of the patient while, at the same time, a need to respect the privacy of the family. This balance was difficult to achieve. In order to minimise inter-personal conflict, formal carers reported having to maintain a neutral position, maintain a low profile and the perceived need to be ‘seen and not heard’ (similar to ‘holding your tongue’, reported by Dybwik et al. (2011)). As such they had to ‘know their place’ in the family home- as a health care professional as opposed to a guest (as reported by Oresland et al. (2008)).They reported being respectful of the ‘line’ or ‘bubble’- they were expected to fulfil a role and not integrate themselves within the family unit. However, this created discomfort- they often felt rude being in the company of the wider family and not communicating with them. Many had their authority challenged by informal carers (as reported previously by Kirk and Glendinning (1999)) and felt as though they were ‘tiptoeing around’- so as not to upset the volatile, fragile dynamics of the household- perhaps they were mindful of not over-stepping their role or inadvertently bursting the informal carers’ ‘bubble’. Given such relational tension, it is perhaps not surprising that there is such a high turnover of formal carers.

While the triad emphasised the stressful, debilitating nature of such interpersonal conflict, they did report a number of potential remedies. Both patients and informal carers reported a need to be actively involved in the selection process of appointing formal carers. For example, as William, one of the patients, and Sharon, one of the informal carers commented:-

I don’t have any control over who they hire. I’m not involved in the interview process. If there’s a vacancy in my team, I’ve got nothing to do with the interview. I’m not involved in that process of the interviews and who they select. I’ve got to trust whoever the package, the manager of the package is that he knows me well enough to pair me with the right person. He can have all the skills in the world but I would like to think that the package manager would know me well enough to say ‘Right that person, they might be good’ (William)

It was just all the more stressful because we didn’t have the right to have control over what was happening and who came in. Someone else was organising it and it just wasn’t working, clearly. A lot of the people were just purely, they were just incompetent (Sharon)

This involvement of the triad was considered a vital opportunity to ‘vet’ the formal carers for ‘fit’. This was important to facilitate trust, provide reassurance of competency and to optimise the psychosocial environment. The formal carers discussed the positive outcomes in finding such a ‘fit’- a natural, healthy and tailored relationship which met all the needs of the triad.

There are a number of clear messages from this study. The design utilises the best of individual data collection and idiographic data analysis. However, this has been combined with a parallel focus upon the juxtaposition of shared findings across the three constitutive participant groups. This has enabled a close-grained in-depth psychological examination of a systemic problem and has enabled the research team to imagine system level interventions.

We have shown that fragmented, hostile communication and collaboration between patients and formal carers is unacceptable and only creates a negative psycho-social environment for the triad. It would appear that inter-personal conflict results in intra-personal conflict and vice versa. Psychological interventions aimed at facilitating the setting of clear, workable boundaries and ongoing support to modify/sustain them over time, are key. Awareness of the importance of a ‘care bubble’ model for care may aid the retention of independence for patients and provide clear boundaries for formal carers to work within. Open communication regarding boundaries for retaining privacy in the early stages of transition to home care, are important. A gradual ‘phasing out’ of the informal carers responsibilities as the formal care team enter the home may facilitate a transition phase for informal carers, build rapport between carers and facilitate trust. This in turn, may improve communication between carers and facilitate acceptance of them within the home. Less resistance and resentment from informal carers may improve the psychosocial experience for formal carers, improving continuity of care, reducing turn-over of staff and optimising the success of the care environment in all respects. Finally, it is important to acknowledge these findings when developing clinical guidelines for ventilation dependent patients. Patient and family carers should be consulted in the recruitment of formal carers in order to find a ‘fit’ – this may minimise relational conflict in the dyad. A multi-perspective approach must be considered to care- it is just not just the needs of the patient that is to be considered- but the intra- and inter-personal aspects of the triad as a whole.

We conclude with a consideration of the limitations of the current study. First, the sample must be pondered. Like much of the extant carer literature (Brannen & Petite, 2008; Dickson et al., 2012; Tang, 2009), the majority of carers were female (providing care for male patients). Future research could consider gender dynamics within the carer perspective. Patients were on the whole, middle-aged or over (mean 44 years) and time since injury was considerable. Although we did not find differences between individual participants on the basis of age, time since injury or the nature of their carer relationship (e.g. spouse, parent, sibling) here, future work could focus on younger patients, particularly those within the early stages of post-hospital discharge as this may create a different series of tensions, strains and relational conflicts to be addressed. We propose that the previous nature of the patient-informal carer relationship (whether it be spouse, sibling or parent) could also influence role adjustment, identity change and relational conflict in assuming the role of informal carer. The nature of relationship change was not captured here in the present study but should be explored further in future work. As we recruited a purposive sample rather than a strictly representative sample, the results represent this particular group of individuals living with mechanical ventilation post SCI in the UK only. They are not, therefore, representative of the experiences of all individuals living with mechanical ventilation and/or SCI more generally.

Moreover, geographically, the patients were widely dispersed across Scotland. Interviews, therefore, were conducted with all three members of the triad on the same day. Although the interviews were conducted individually and in private, there were occasions where the participants would whisper in fear of others overhearing their narrative. This may have inhibited a full, honest disclosure of their intra- and inter-personal experiences. Future research could consider conducting telephone interviews with formal carers (out-with working hours) and interviewing informal carers out-with the home environment. Due to the very nature of the patient’s condition (and their dependency on the ventilator), interviews were considerably shorter than is typical for IPA (maximum of 45 min). Patients were also reluctant to discuss anything considered too sensitive in fear of becoming upset (and potentially impairing their breathing). The researchers refrained for pursuing such lines of questioning as a direct response to their NHS contract. Again, this may have inhibited disclosure of material. Finally, this article only represents this particular group of participants’ experiences. Thus the findings are suggestive as opposed to conclusive and cannot be generalised to the wider HMV and/or SCI population. We suggest that longitudinal research capturing the establishment of boundaries and its associated interpersonal challenges over time, is warranted. This should increase an understanding of specific conflicts, tensions and stressors within the triad at distinct points in time. This would promote a more holistic understanding of the relational challenges facing this group and would have potential to influence health education, health care policy and future practice of health care providers informed by, and tailored towards, the needs of the triad themselves. Other complimentary research approaches such as observational methods and ethnographic work would be a fine compliment to the work presented herein.
